# Shikonin Attenuates Cochlear Spiral Ganglion Neuron Degeneration by Activating Nrf2-ARE Signaling Pathway

**DOI:** 10.3389/fnmol.2022.829642

**Published:** 2022-02-24

**Authors:** Hongjie Du, Xuanchen Zhou, Lei Shi, Ming Xia, Yajie Wang, Na Guo, Houyang Hu, Pan Zhang, Huiming Yang, Fangyuan Zhu, Zhenxiao Teng, Chengcheng Liu, Miaoqing Zhao

**Affiliations:** ^1^Department of Otolaryngology, Shandong Provincial Hospital Affiliated to Shandong First Medical University, Jinan, China; ^2^Qilu Pharmaceutical Co., Ltd., Jinan, China; ^3^Department of Otolaryngology, Shandong Provincial Hospital, Cheeloo College of Medicine, Shandong University, Jinan, China; ^4^Central Laboratory, Shandong Provincial Hospital Affiliated to Shandong First Medical University, Jinan, China; ^5^Department of Pathology, Shandong Provincial Hospital Affiliated to Shandong First Medical University, Jinan, China

**Keywords:** shikonin, spiral ganglion cells, auditory nerve damage, Nrf2-ARE, ouabain

## Abstract

The molecular mechanisms that regulate the proliferation and differentiation of inner ear spiral ganglion cells (SGCs) remain largely unknown. Shikonin (a naphthoquinone pigment isolated from the traditional Chinese herbal medicine comfrey root) has anti-oxidation, anti-apoptosis and promoting proliferation and differentiation effects on neural progenitor cells. To study the protective effect of shikonin on auditory nerve damage, we isolated spiral ganglion neuron cells (SGNs) and spiral ganglion Schwann cells (SGSs) that provide nutrients *in vitro* and pretreated them with shikonin. We found that shikonin can reduce ouabain, a drug that can selectively destroy SGNs and induce auditory nerve damage, caused SGNs proliferation decreased, neurite outgrowth inhibition, cells apoptosis and mitochondrial depolarization. In addition, we found that shikonin can increase the expression of Nrf2 and its downstream molecules HO-1 and NQO1, thereby enhancing the antioxidant capacity of SGNs and SGSs, promoting cells proliferation, and inhibiting cells apoptosis by activating the Nrf2/antioxidant response elements (ARE) signal pathway. However, knockdown of Nrf2 rescued the protective effect of shikonin on SGNs and SGSs damage. In addition, we injected shikonin pretreatment into mouse that ouabain-induced hearing loss and found that shikonin pretreatment has a defensive effect on auditory nerve damage. In summary, the results of this study indicate that shikonin could attenuate the level of oxidative stress in SGNs and SGSs through the Nrf2-ARE signaling pathway activated, induce the proliferation and differentiation of SGNs, and thereby improve the neurological hearing damage in mice. Therefore, shikonin may be a candidate therapeutic drug for endogenous antioxidants that can be used to treat neurological deafness.

## Introduction

Sensorineural hearing loss is a sensorineural disorder that affects human physical and mental health and quality of life. The pathological feature of sensorineural hearing loss is corti organ dysfunction (Chen et al., 2021; Fu et al., 2021a; He Z. H. et al., 2021) and spiral ganglion neuron degeneration subsequently (White et al., 2000; Liu et al., 2019a). Spiral Ganglion Neuron (SGN), as the first afferent neuron in the auditory pathway, transmits mechanical sound signals received from the outside world from hair cells to cochlear sensory neurons for processing, thereby establishing the bridge between physics and perceptual world of sound (Reijntjes and Pyott, 2016; Tan et al., 2019; Wei et al., 2021). Drugs, noise, and hypoxia can easily cause SGN damage (Guo et al., 2021a; Liu et al., 2021), which can lead to hearing loss, age-related hearing loss and even auditory neuropathy (Waqas et al., 2017; Liu et al., 2019b). Studies have shown that because SGNs cannot regenerate, the loss of cochlear signals can lead to irreversible hearing damage (Liu et al., 2019a; Ding et al., 2020). Thus, recently many studies have focusd on the regeneration of SGN via neural stem cells (Guo et al., 2021b; He Z. et al., 2021). Spiral ganglion Schwann cells (SGSs) are glial cells responsible for myelination in the peripheral nervous system (Wise et al., 2017; Meas et al., 2018). When the spiral ganglion cells are damaged, it can provide the spiral ganglion cells with the nutrient factors needed for growth, help the damaged neurons to repair, and promote the growth of neurites (Wang et al., 2009; Provenzano et al., 2011). The existence of SGSs can greatly improve the survival rate of SGNs and the repair ability of neurons (Pettingill et al., 2008; Provenzano et al., 2011; Lei and Tang, 2017). Therefore, Schwann cells play an irreplaceable role in the development of the cochlear nervous system, especially in the recovery and protection of the damaged nervous system.

Exogenous factors such as ototoxic drugs and noise can induce SGNs oxidative stress and mitochondrial dysfunction, resulting in abnormal SGNs proliferation and differentiation, apoptosis or programed cells death (He et al., 2016; Sun et al., 2016). These cellular processes and the resulting SGNs dysfunction and degradation are the most important causes of hearing loss. The loss of function and neurological degeneration of SGN impairs the transmission of nerve signals that carry sound information. The use of therapeutic drugs with ototoxic side effects, such as aminoglycoside antibiotics (Selimoglu, 2007), ouabain (Zhang et al., 2017), and cisplatin (Liu et al., 2019b), can cause the damage or loss of mammalian SGNs, which can lead to permanent deafness. Ototoxic drugs can cause cochlear neurons to produce reactive oxygen species (ROS), which promotes cell apoptosis in turn (Tabuchi et al., 2011). The oxidative stress response in SGNs is triggered by oxidative imbalance, which leads to oxidative damage, which in turn causes hearing loss (Kim et al., 2020). Currently, cochlear implants can be used to restore the hearing of patients with severe sensorineural hearing loss (Buchman et al., 2020). The survival and protection of SGNs are necessary for cochlear implantation and patient hearing recovery (Guo R. et al., 2019; Guo et al., 2021c). SGSs can proliferate during neuronal regeneration after injury, and their cellular proliferation, differentiation, and myelination depend on interactions with axons (Kobayashi et al., 2012). The identification of signals that mediate SGN-SGS cells interactions is relevant not only to normal auditory nerve development, but also to its maintenance and possible repair (Castelnovo et al., 2017). If the degradation of SGNs can be slowed, the effect of cochlear implants will be further optimized. Therefore, preventing or reducing the damage and degeneration of cochlear SGN is essential for the recovery of sensorineural hearing loss.

Noise-induced hearing loss, ototoxic drugs, and age-related factors are always related to the increase in autophagy and oxidative stress in the mouse cochlea SGN, which ultimately leads to SGN cell apoptosis (Fu et al., 2021b; Zhang et al., 2021). Studies have shown that antioxidant drugs can reduce SGN loss and hearing damage, indicating that the oxidative stress level of SGN can affect hearing status (Guo et al., 2021a). Shikonin is the main component of the extract of the Chinese herbal medicine comfrey. It has anti-bacterial, anti-tumor, anti-oxidation, anti-inflammatory, and anti-apoptosis effect (Gwon et al., 2020; Wang et al., 2020; Zhou et al., 2020). Research data has shown that shikonin induces antioxidants and protects cells from oxidative stress by reducing the level of free radicals and cytotoxic substances in cell (Guo H. et al., 2019; Gwon et al., 2020). Recently, studies have shown that shikonin has neuroprotective activity against traumatic or ischemic neuronal damage regulated by oxidative stress and apoptosis pathways (Wang et al., 2010). Previous studies have also proved that shikonin can reduce spinal cord ischemia-reperfusion injury by improving the level of mitochondrial oxidative stress (Wang et al., 2010). However, there is no report on the anti-apoptosis and antioxidant effects of shikonin on SGNs caused by ototoxic drugs.

Nrf2 is a redox-sensitive transcription factor that plays a key role in the regulation of oxidative stress (Ma, 2013). Under oxidative stress conditions, Nrf2 accumulates in the nucleus and activates the expression of antioxidant response elements (ARE) dependent genes, including heme oxygenase 1 (HO-1), superoxide dismutase (SOD), and peroxidase, catalase (CAT), glutathione synthase (GS), glutathione reductase (GR), glutathione peroxidase (GPX) and glutathione-*S*-transferase (GST) (Albarakati et al., 2020). It is reported that age-related hearing loss is accelerated in SOD1 knockout mice, and SOD1 knockout mice are more prone to hearing loss when affected by noise (Ohlemiller et al., 1999). Tempol (a SOD simulator) can prevent noise from causing damage to hearing (Minami et al., 2007). In addition, HO1 can also effectively protect the inner ear from damage caused by oxidative stress (Kim et al., 2006). There is enhanced evidence that the regulation of the Nrf2/ARE signaling pathway plays an important role in neurological deafness.

In this study, we used the ototoxic drug ouabain to establish a mouse model of neurological deafness and observe the damage to the hearing and SGCs of the mice. To find the pathogenic mechanism of ouabain’s ototoxicity, we isolated and cultured mouse SGNs and SGSs *in vitro* and detected that ouabain inhibits SGNs proliferation and neurite growth, and promotes cell mitochondrial depolarization. The increase in the level of oxidative stress in SGNs eventually leads to an increase in the level of cell apoptosis, which in turn leads to the occurrence of neurological deafness. Shikonin, the main component of comfrey extract, can activate the Nrf2/ARE signaling pathway, thereby alleviating ouabain-induced increase in the level of oxidative stress in SGNs, cells apoptosis and hearing loss in mice. It provides a new choice of antioxidant drugs for the treatment of neurological deafness.

## Materials and Methods

### Animals

All animal experiments were approved by the Ethics Committee of Shandong Provincial Hospital Affiliated to Shandong First Medical University Permit Number: No.2020-422 and were performed in accordance with the relevant guidelines and regulations. Keep animals in a controlled environment with constant temperature and humidity, and provide enough sterile food and water.

### Reagants

Ouabain (Cat. No. S4016) was obtained from Selleck. Shikonin (Cat. No. 517-89-5) and ML385 (Cat. No. 846557-71-9) were purchased from Merck (Billerica, United States). Rabbit polyclonal anti-Nrf2 antibody (Cat. No. sc-173), anti-NeuN (Cat. No. ab177487), anti-Tuj1 (Cat. No. ab52623) and anti-Nestin (Cat. No. ab254048) was from Abcam (Cambridge, United Kingdom). Rabbit polyclonal anti-LC3B antibody (Cat. No. A19665), anti-Bax (Cat. No.A19684), anti-Casepase3 (Cat. No. A11319) and anti-Actin (Cat. No. AC026) was from Abclonal (Wuhan, China), and rabbit polyclonal anti-Bcl2 antibody (Cat. No. 12789-1-AP) and anti-SQSTM1 antibody (Cat. No. 18420-1-AP), was from Proteintech (Wuhan, China). The primary mouse anti-S100 antibody (Cat. No. S2532) obtained from Sigma (St. Louis, United States). Rabbit monoclonal anti-VGLUT1 (Cat. No. #12331), anti-GAT1 (Cat. No. #37342) was from Cell Signaling Technology (Boston, MA, United States). Mouse polyclonal anti-HO-1 antibody (Cat. No. 66743-1-Ig) and anti-NQO1 antibody (Cat. No. 67240-1-Ig) was from Proteintech (Wuhan, China).

### Primary Explants and Cells Isolation Culture of Mouse Cochlear Spiral Ganglion

Decapitated inner cochlea of P3 C57BL/6 J WT mice and placed in pre-cooled PBS. Then, the volute, spiral ligament and stria vascularis are removed. Finally, the removed spiral ganglion tissue explant was placed on a glass cover slip, which was pre-coated with CellTaK (Corning, Cat. No.354240). Add DMEM/F12 (Gibco, 11,330,032), which contains epithelial growth factor (EGF, 20 ng/ml), N2 additives (Gibco, catalog number 17502-048) and 1% ampicillin (Sigma-Aldrich, St. Louis, United States) in the petri dish. We then obtain spiral ganglion cells. After the cells were cultured for 48 h, 10 μM cytarabine was added for treatment, and then the cells were replaced with a medium without N2 and serum, and the culture was continued to obtain Schwann cells. After the cells were cultured for 48 h, 10 μM cytarabine was added for treatment, and then the cells were replaced with a medium without N2 and serum, and the culture was continued to obtain Schwann cells.

Transfer the dissected spiral ganglion explants into a sterile tube equipped with pre-cooled D-hanks. After washing twice with D-hanks, add a digestion solution containing 0.25% trypsin and 0.001% DNase. After digestion in a 37°C incubator for 20 min, add a serum-containing medium to terminate the digestion, gently pipette to form single cells, inoculate them into a culture dish containing the above medium, and cultivate in a 37°C and 5% CO_2_ incubator.

### *In vivo* Drug Treatments

All mouse experiments were performed using 8-month-old female C57BL/6 WT mice *in vitro*. As stated in the text: ouabain (3 mM) was injected for 5 consecutive days, recovered for 3 days, and then injected for 5 consecutive days; Shikonin (10 mM) was injected 3 days in advance for pretreatment, and ML385(30 mg/Kg) was injected 1 day before for pretreatment; Control mice were replaced with the same dose of saline at the same time point.

### Apoptosis and Cell Cycle Analysis

The cell cycle and apoptosis were analyzed by flow cytometry. PI staining assesses cell cycle distribution. Seed the cells in a 6-well tissue culture plate (40,0000 cells/well). After 24 h of culture, drugs were added for another 24 h, and the cells were collected and added RNase A solution, incubate the cells at 37°C. Finally, add 400 μL of PI and incubate at room temperature for 30 min. The DNA content was detected by flow cytometry, and the percentage of cells in G1, S, and G2/M phases was analyzed.

According to the manufacturer’s instructions, the AnnexinV-FITC/PI apoptosis detection kit was used to evaluate the apoptosis rate. After cells treatment, the cells were collected in binding buffer. Then, Annexin V-FITC and PI were added and incubated at room temperature in the dark. The level of apoptosis was analyzed by flow cytometry.

### Oxidative Stress Level Detection

Spiral ganglion neuron cells were inoculated into a 6-well tissue culture plate (40,0000 cells/well), after 24 h of culture, drugs were added for another 24 h. According to the manufacturer’s instructions, use a reactive oxygen detection kit (Cat. No. S0033S, Beyotime) to detect ROS levels, a total SOD activity detection kit (WST-8 method, Cat. No. S0101S, Beyotime) to detect SOD levels, and Lipid Peroxidation (MDA) Assay Kit (Cat. No. K739-100, Biovision) detects MDA levels, and total glutathione (GSH) detection kit (Cat. No. S0052, Beyotime) detects GSH levels.

### Auditory Brainstem Response Measurements

The hearing status of mice was detected by the auditory brainstem response (ABR) threshold. In brief, animal were anesthetized with pentobarbital intraperitoneal injection (8.4 mg/100 g). Place the mouse on a constant temperature pad and keep the body temperature at 37°C. Insert electrodes on the top, subcutaneously at the outer auricle, and back of the mouse’s skull. RZ6 workstation and BioSig software (Tucker-Davis Technologies, Inc., Alachua, FL, United States) were used for ABR data collection and generation. At each sound level, 512 responses are sampled and averaged. The minimum detectable threshold of all ABR waves is determined by gradually reducing the stimulus intensity from 90 dB SPL to 10 dB SPL, so as to obtain the ABR threshold of each animal.

### Transfection and Gene Expression Silencing

Small hairpin RNA (shRNA) was used to silence Nrf2 gene expression. Nrf2 siRNAs were obtained from Invitrogen (Nrf2-si-1: siRNA ID 68238; Nrf2-si-2: siRNA ID 156499; and Nrf2-si-3: siRNA ID 156501). For effective gene silencing, siRNAs were transfected twice. In short, according to the manufacturer’s instructions, use jetPRIME ^®^ Transfection Agent (Polyplus) to transfect cells with 30 nM negative control (NC) siRNA and Nrf2-siRNA. After 24 h of incubation, a second transfection was performed.

### Cell Proliferation Assay

MTT assay was performed to measure the cell proliferation. Spiral ganglion cells were seeded in a 96-well plate at 10,000 cells/well in five replicates. After 24 h, the cells were pretreated with DHC and SHI for 2 h, and then added with ouabain for another 22 h. Then discard the supernatant and replace with fresh serum-free medium. According to the instructions of the MTT Proliferation Assay Kit (ab211091), add MTT to the cell culture medium and incubate it in a 37°C incubator. Subsequently, MTT solvent was added to the cell culture medium, and the 96-well plate was shaken for 5 min in the dark, and the absorbance was read at OD = 570 nm, and then the cell proliferation was evaluated.

### Analysis of Mitochondrial Membrane Potential

JC-1 assay kit (C2006, Beyotime) was used for JC-1 analysis according to the manufacturer’s instructions. Brief, the cultured cells were washed once with PBS, and then fresh cell culture medium was added. Add an equal volume of JC-1 staining working solution to the culture solution, mix well, put it into the cell culture incubator, and incubate at 37°C for 20 min. Then discard the supernatant and wash twice with JC-1 staining buffer. Collect the processed cells, and then use the flow cytometer to detect and analyze.

### Immunofluorescence

The spiral ganglion primary tissues or cells were cultured on glass coverslips coated with gelatin and incubated at 37°C for 24 h, and then treated with ouabain for another 24 h. The cell supernatant was discarded and washed twice with PBS, then fixed with 4% paraformaldehyde (PFA) for 15 min, and then washed with PBS. After permeating the cell membrane with 0.2% TritonX-100, it was blocked with goat serum. Add the primary antibody and incubate overnight at 4°C. The next day, the corresponding fluorescent secondary antibody was added, and after incubating for 1 h at room temperature, the nuclei were stained with DAPI (Gen-View Scientific Inc., Galveston, TX, United States) and mounted. Finally, the cells were imaged and observed using an upright fluorescent confocal microscope (LSM 700, Zeiss). The measurement of nerve cell dendrites is the straight-line distance from the center of the nerve cell body to the tip of the longest dendrite.

### Quantitative Real-Time Polymerase Chain Reaction

RNeasy Micro Kits (Qiagen) was used to extract total RNA from mouse spiral ganglion cells or tissues according to the manufacturer’s instructions. Subsequently, reverse transcription was performed using a reverse transcription kit (Invitrogen), and the cDNA obtained by reverse transcription was used as a template to perform real-time quantitative PCR using the SYBR ^®^ Premix Ex Taq™ system (Perfect Real Time, Thermo Fisher Scientific, Waltham, MA, United States). The primer sequences used in the study are shown in Table 1. In order to obtain the best sensitivity and specificity, the PCR reaction set is set to: The initial cycle of 95°C for 30 s, followed by 40 cycles of 95°C for 5 s, 58°C for 20 s, and the final cycle is 95°C for 15 s, 62°C for 1 min, and 95°C for 15 s. The negative control is a sample without template and processed in the same procedure. All reactions were performed in triplicate. Melting curve analysis was used to verify the specificity of amplification. The relative expression of mRNA of each gene was calculated by the 2^–ΔΔCT^ method.

**TABLE 1:** Sequences of primers used for quantitative real-time polymerase chain reaction (qRT-PCR).

Gene	Sense primer	Anti-sense primer	Lengths
*Nrf2*	5′-ATTCTGCTTTCATAG CAGAG -3′	5′-ACTCGTGTTCAGTGA AATG -3′	196 bp
*HO-1*	5′-TACCTGGGTGACCT CTCA-3′	5′-ATAGAGCTGTTTGA ACTT-3′	138 bp
*NQO1*	5′-TAAGGAAGGACGCC TGAG-3′	5′-AGCAGTCTCTCAAAC-3′	134 bp
*VGLUT1*	5′-TTGGCTTTGCCATTG TGGC-3′	5′-GACTCCGTTCTAA GGGA-3′	173 bp
*GAT1*	5′-TTGGACTGGAAAG GTGGT-3′	5′-ACAGCTTTCGGAAGTT-3′	136 bp
β*-actin*	5′-CTCCATCCTGGCCTCG CTGT-3′	5′-GCTGTCACCTTCACCG TTCC-3′	268 bp

### Western Blotting

Dissect mouse spiral ganglion tissue or cultured cells, wash with pre-cooled PBS, add RIPA lysis buffer containing protease inhibitor Cocktail (Merck, Millipore 539131-1VL), and lyse on ice. After centrifugation at 4°C, the supernatant was collected and the protein concentration was determined with the BCA Protein Assay Kit (P0011, Beyotime). Add protein loading buffer for sample preparation, then perform polyacrylamide gel electrophoresis (PAGE), and transfer the protein to PVDF membrane. After blocking with skimmed milk powder, add the corresponding primary antibody and incubate overnight at 4°C. The next day, add the corresponding secondary antibody and incubate at room temperature for 1 h. The signal band was then detected by the chemiluminescent detection method (Amersham ECL).

### Statistical Analysis

In the statistical analysis of the data, GraphPad Prism9 software was used. All data are shown as the mean ± SD (standard error of the mean) from at least 3 independently performed experiments, and ‘*n*’ represents the number of samples. A two-tailed unfit Student’s *t*-test was used to determine statistical significance of two sets of data. For statistical analysis of multiple sets of data, two-way analysis of variance and post-event Student–Newman–Keuls test are used for significance analysis. And *P* < 0.05 is considered statistically significant.

## Results

### Ouabain Damages the Hearing of Mice and Activates Spiral Ganglion Schwann Cells Apoptosis and Autophagy

**FIGURE 1:**
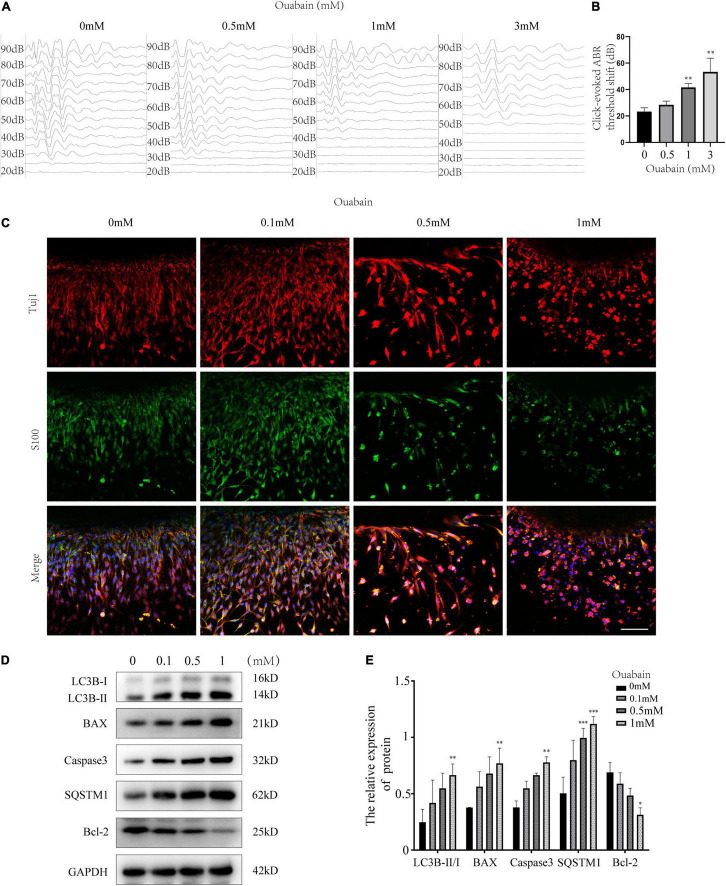
Ouabain damages the hearing of mice and activates SGSs apoptosis and autophagy. **(A)** Ouabain drugs of different concentrations (0.5, 1.0, and 3 mM) were treated for 7 days, and the auditory brainstem response waveforms of the mice were detected (*n* = 3). In the control group, the mice were injected with the same dose of saline. **(B)** Statistical histogram of mouse hearing threshold. **(C)** Immunofluorescence observed the changes in cell morphology of SGSs explants treated with different concentrations of ouabain (0.1, 0.5, and 1 mM) for 24 h. Red fluorescence represents Tuj1 staining, green fluorescence represents S100 staining, and blue fluorescence represents nuclear (DAPI) staining, bar = 50 μm. **(D)** After SGNs were treated with different concentrations of ouabain drugs (0.1, 0.5, and 1 mM) for 24 h, cell proteins were collected, and Western Blot was used to detect cell apoptosis and autophagy protein level changes. **(E)** Statistical histogram of relative protein expression levels. *P < 0.05, ^**^P < 0.01, ^***^P < 0.001.

To evaluate the damage effect of ouabain on the hearing of mice, we used different concentrations of ouabain to continuously inject 8-week-old C57BL/6 mice for 5 days, and detect the changes in the hearing threshold of the mice on the 7th day. Zhang et al. (2017) showed that ouabain was dose-dependent on spiral ganglion neurons and Schwann cells in mice, and the optimal dose ranged from 0.5 mM to 3. Therefore, we chose 0–3 mM ouabain to stimulate spiral ganglion neurons and Schwann cells in mice. The hearing brainstem response (ABR) results of mice showed that with the increase of ouabain concentration, the hearing threshold of mice gradually increased (Figures 1A,B). Ouabain has obvious damage to the hearing of mice.

The existence of SGSs is essential to the maintenance of SGNs function, and studies have shown that SGN dysfunction and degeneration are the most important causes of hearing loss (Chai et al., 2017). In order to observe the damage effect of ouabain on mouse spiral ganglion tissue, we dissect and culture SGSs explants from P3 newborn mice *in vivo*, then treat them with different concentrations of ouabain. Considering that cells cultured *in vitro* are more sensitive to drugs than *in vivo*, the concentration of drug treatment is reduced accordingly. S100 is highly expressed in mature Schwann cells (Fregoso and Hoover, 2012), so we labeled Schwann cells with S100. Immunofluorescence results showed that, compared with the control group, as the concentration of ouabain increased, the SGSs explants gradually appeared cell death, and the morphological damage was gradually serious (Figure 1C). Subsequently, we examined the changes in the level of apoptosis in mouse SGSs explants. The expression of apoptosis marker proteins Caspase 3 and Bax was significantly increased, while the expression of anti-apoptotic protein Bcl-2 was significantly decreased (Figures 1D,E). In addition, the autophagy marker proteins LC3B and SQSTM were also tested, and the results showed that the level of autophagy was also significantly increased (Figures 1D,E). The above results suggest that ouabain can cause hearing damage in mice, and spiral ganglion damage plays an important role in this process.

### Ouabain Affects the Differentiation of Spiral Ganglion Neuron Cells Into Glutamatergic and GABAergic Neuronal Groups

**FIGURE 2:**
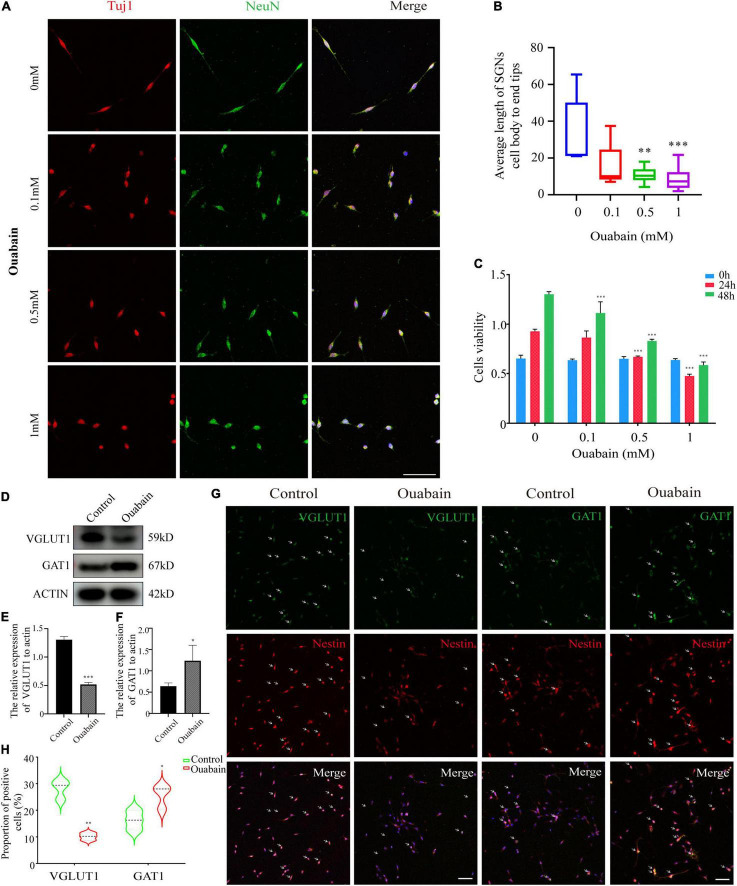
Ouabain affects the differentiation of SGNs into glutamatergic and GABAergic neuronal groups. **(A)** After separating the SGNs, they were treated with different concentrations of ouabain (0.5, 1.0, and 3 mM) for 24 h, and the cell morphology changes were observed by immunofluorescence. Red represents Tuj1 staining, green fluorescence represents NeuN staining, blue fluorescence represents nuclear (DAPI) staining, bar = 50 μm. **(B)** Statistics of the average length of SGNs cell body to the apex of the longer synapse after treatment with different concentrations of ouabain. **(C)** Cell viability (0, 24, and 48 h) after treatment with different concentrations of ouabain was detected by MTT. **(D)** The expression of VGLUT1 and GAT1 proteins was detected after 24 h of treatment with 0.5 mM ouabain. **(E)** Statistical histogram of relative expression of VGLUT1 protein. **(F)** Statistical histogram of the relative expression of GAT1 protein. **(G)** Immunofluorescence observation of the number of VGLUT1 and GAT1 positive cells after ouabain treatment. Red fluorescence represents Nestin staining, green fluorescence represents VGLUT1 or GAT1 staining, blue fluorescence represents nuclear (DAPI) staining, white arrows represent positive cells, bar = 50 μm. **(H)** Statistical histogram of the proportion of VGLUT1 and GAT1 positive cells. *P < 0.05, ^**^P < 0.01, ^***^P < 0.001.

In order to observe the effect of ouabain on the cell morphology of spiral ganglion cells (SGNs) more clearly, we separated spiral ganglion cells by enzymatic hydrolysis and performed immunofluorescence staining with NeuN, a specific marker for screening and identifying neurons (Duan et al., 2016). The results showed that with the increase of ouabain concentration, SGNs cells gradually changed from elongated to elliptical, and the synapse length was significantly shortened (Figures 2A,B). Furthermore, the cellular viability of SGNs decreased and the level of apoptosis increased with increasing ouabain concentration (Figure 2C and Supplementary Figure 1). Glutamatergic and GABAergic neurons in the cochlear nerve cells secrete excitatory and inhibitory neurotransmitters, respectively, to regulate the transmission of nerve impulses in the cochlea. We tried to explore the effect of ouabain on the transmission of nerve impulses in SGNs by detecting the effect of ouabain on the differentiation of glutamatergic and GABAergic neurons. Western blot results showed that the addition of ouabain inhibited the expression of glutamate transporter 1 (VGLUT1) and promoted the expression of GAT1 protein (GABAergic neurons) (Figures 2D–F). The rate of VGLUT1 positive cells was also significantly lower than that of GAT1 positive cells (Figures 2G,H). The above results suggest that ouabain can damage the morphology and differentiation of spiral ganglia and inhibit neuronal excitation.

### Shikonin Can Relieve Ouabain’s Damage to the Growth and Proliferation of Spiral Ganglion Schwann Cells

**FIGURE 3:**
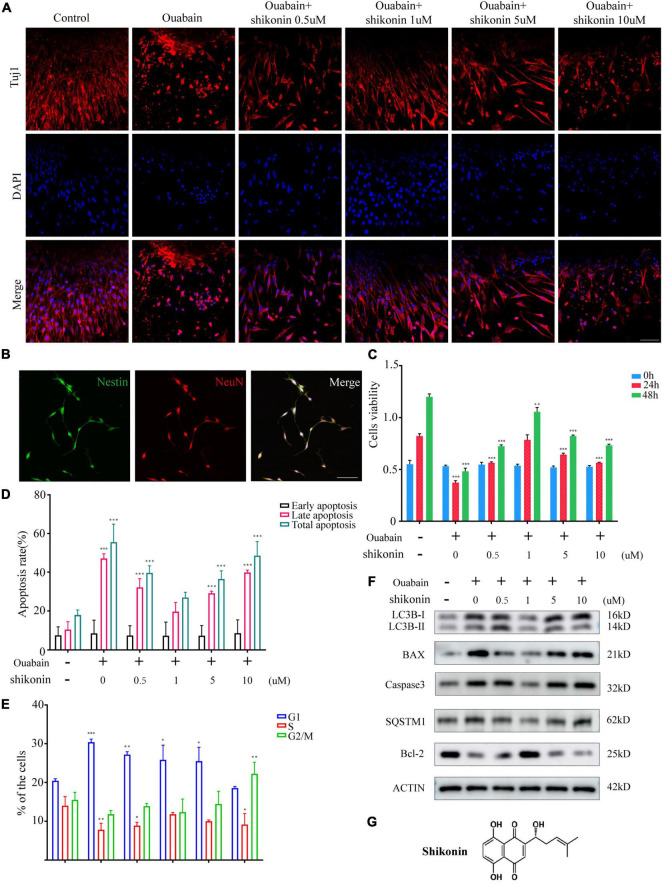
Shikonin can relieve ouabain’s damage to the growth and proliferation of SGNs and SGSs. After pretreatment of SGSs explants with different concentrations of shikonin (0.5, 1, 5, and 10 μM) for 2 h, 0.5 mM ouabain continued treatment for another 22 h. **(A)** The morphology of SGSs was observed by immunofluorescence. Red fluorescence represents Tuj1 staining, blue fluorescence represents nuclear (DAPI) staining, bar = 50 μm. **(B)** Nestin and NeuN perform nerve cell labeling. Among them, red represents NeuN staining, green fluorescence represents Nestin staining, and blue fluorescence represents nuclear (DAPI) staining, bar = 50 μm. **(C)** After treatment with shikonin and ouabain, cell viability detection (0, 24, and 48 h). Flow cytometry was used to detect the changes of apoptosis **(D)** and cell cycle **(E)** after treatment with shikonin and ouabain. **(F)** The effects of shikonin pretreatment on apoptosis and autophagy were detected by Western blot. **(G)** Structural diagram of shikonin. *P < 0.05, ^**^P < 0.01, ^***^P < 0.001.

Shikonin is an extract component of comfrey (Figure 3G). A large number of studies have shown that shikonin has obvious anti-inflammatory, anti-oxidant and anti-apoptotic effects. We tried to explore whether shikonin has a protective effect on cell death caused by ouabain. We pretreated the SGSs explants with ouabain with a gradient concentration of shikonin, and then observed the cell morphology. Immunofluorescence results showed that as the concentration of shikonin increased, the cell damage of SGSs explants was reduced after ouabain treatment. However, when the concentration of shikonin exceeded a certain limit, the protective effect on the cells gradually disappeared (Figure 3A). We speculate that the effect of shikonin may be bifacial, with low concentrations promoting cell survival and high concentrations inhibiting cell growth. In this regard, we isolated SGNs to detect the effect of different concentrations of shikonin on cell survival and growth. In addition to NeuN, we used Nestin, a neural stem cell marker specifically expressed in adult mammalian neural stem cells known to support mitotic activity (Ernst and Christie, 2006), to co-identify and label spiral ganglion cells (Figure 3B). Subsequently, the cell viability, cell cycle and apoptosis of SGNs stimulated by ouabain pretreated with different concentrations of shikonin were tested. The results showed that compared with the pretreatment group without shikonin, shikonin at a concentration of 1 μM can increase the cell activity and cell cycle of SGNs inhibited by ouabain, and increase the proportion of S-phase cells (Figures 3C,E). At the same time, it reduces the proportion of late apoptotic cells (Figure 3D), and had a protective effect on ouabain-induced apoptosis of SGNs (Figure 3F). The above results suggest that shikonin has a significant protective effect on SGNs cell damage stimulated by ouabain. However, the shikonin concentration has the most obvious protective effect on ouabain damage when appropriate, and the cytoprotective effect of ouabain is reduced when the concentration is lower or too high.

### Shikonin Can Rescue the Effect of Ouabain on the Differentiation of Spiral Ganglion Neuron Cells

**FIGURE 4:**
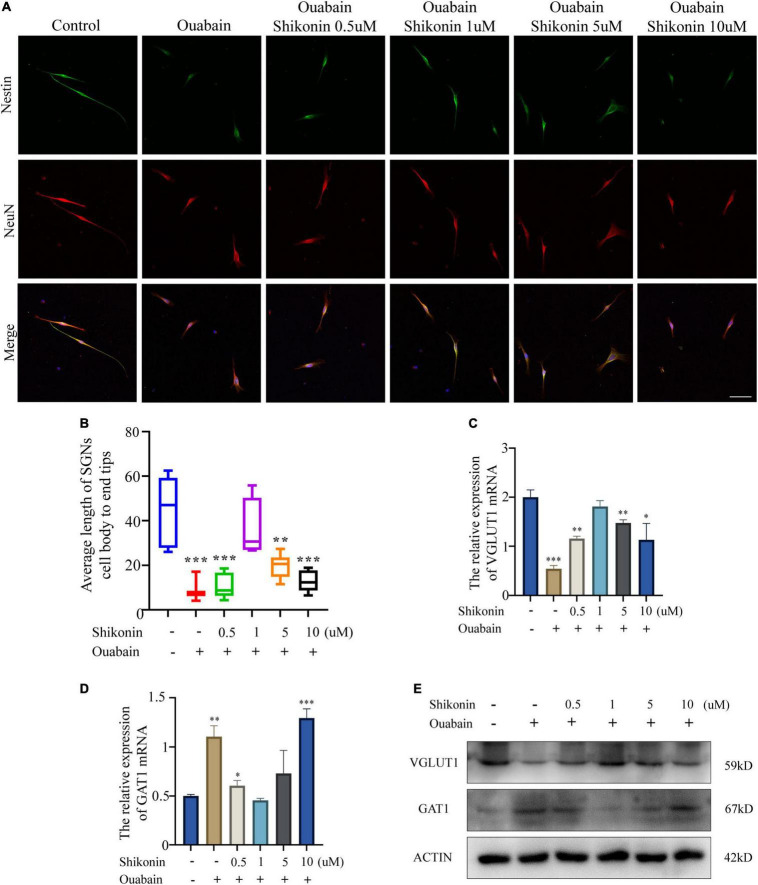
Shikonin can rescue the effect of ouabain on the differentiation of SGNs. After pretreatment of SGNs with different concentrations of shikonin (0.5, 1, 5, and 10 μM) to separate the cells for 2 h, 0.5 mM ouabain continued treatment for another 22 h. **(A)** The morphology of SGNs was observed by immunofluorescence. Red represents NeuN staining, green fluorescence represents Nestin staining, blue fluorescence represents nuclear (DAPI) staining, bar = 50 μm. **(B)** Statistics of the average length of SGNs from the cell body to the apex of the longer synapse in different treatment groups. After collecting cells in different treatment groups, RNA was extracted, and the relative mRNA expression levels of VGLUT1 **(C)** and GAT1 **(D)** were detected by qPCR, and β-antin was used as an internal reference gene. **(E)** The cells of different treatment groups were subjected to protein extraction to detect the expression of VGLUT1 and GAT1 proteins. *P < 0.05, ^**^P < 0.01, ^***^P < 0.001.

Next, in order to explore whether shikonin can alleviate the suppression of nerve impulse transmission in SGNs caused by ouabain, we measured the changes in the dendritic length of SGNs and the distribution of cell differentiation populations after shikonin treatment. The results showed that compared with the non-pretreatment group with shikonin, shikonin can significantly reduce the length damage of SGNs dendrites caused by ouabain, and can better maintain the cell morphology of SGNs (Figures 4A,B). In addition, shikonin pretreatment can significantly improve the decrease of VGLUT1 expression and the increase of GAT1 expression caused by ouabain, promote the differentiation of glutamatergic neurons, and relieve the inhibition of neuronal excitability caused by ouabain (Figures 4C,D). However, the effects of shikonin above also have the same dose-dependent effect.

### Nrf2/Antioxidant Response Elements Signaling Pathway Inhibited by Ouabain Can Be Activated by Shikonin, Reducing Oxidative Stress in Spiral Ganglion Neuron Cells

**FIGURE 5:**
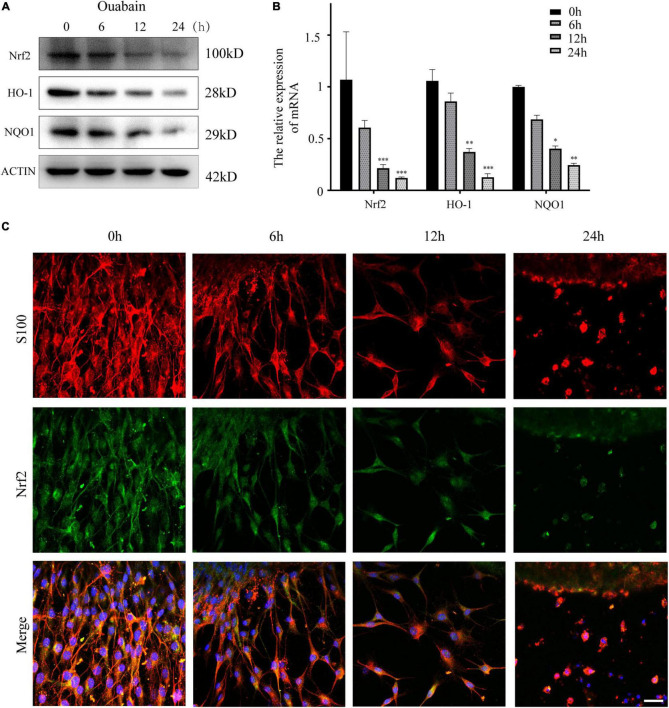
Shikonin can activate the Nrf2/ARE signaling pathway inhibited by ouabain in SGSs. **(A)** After 0.5 mM ouabain was treated with SGSs explants for different times, cell proteins were collected and the protein expression changes of Nrf2/ARE signaling pathway proteins (Nrf2, HO-1 and NQO1) were detected. **(B)** Extract the RNA of each group of cells, and detect the relative expression of Nrf2, HO-1 and NQO1 mRNA by qPCR. **(C)** Immunofluorescence staining to detect the protein expression of Nrf2 after ouabain treatment. Among them, red represents S100 staining, green fluorescence represents Nrf2 staining, blue fluorescence represents nuclear (DAPI) staining, bar = 50 μm. *P < 0.05, ^**^P < 0.01, ^***^P < 0.001.

**FIGURE 6:**
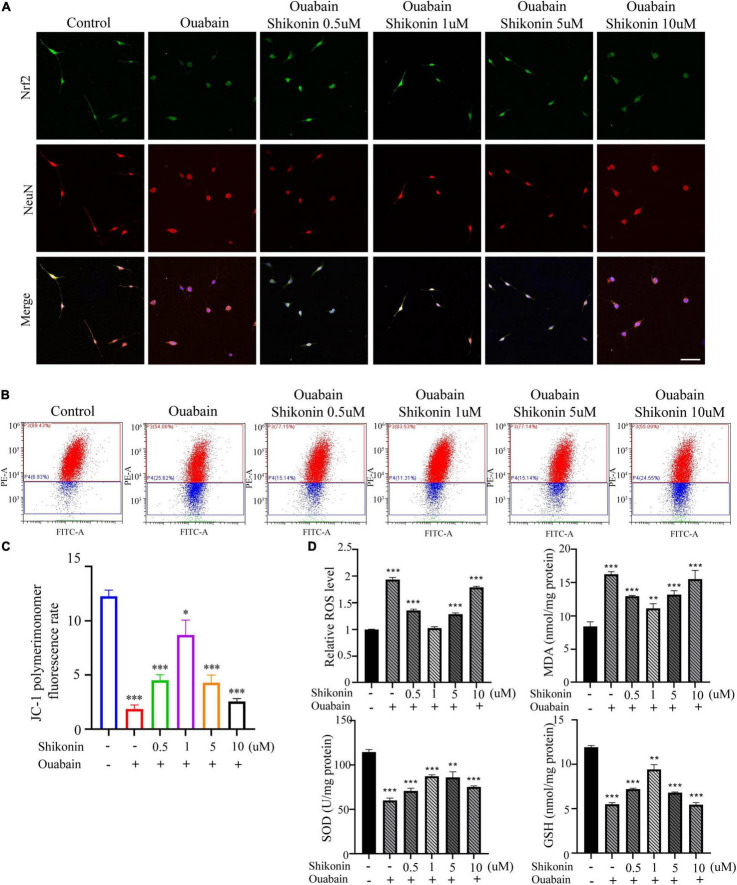
Shikonin can reduce the oxidative stress level of SGNs caused by ouabain. After pretreatment of SGNs with different concentrations of shikonin (0.5, 1, 5, and 10 μM) to separate the cells for 2 h, 0.5 mM ouabain continued treatment for another 22 h. **(A)** After treatment, the morphology of SGNs cells and the expression of Nrf2 protein were observed. Among them, red represents NeuN staining, green fluorescence represents Nrf2 staining, blue fluorescence represents nuclear (DAPI) staining, bar = 50 μm. **(B)** The level of mitochondrial depolarization was measured by flow cytometry to detect the JC-1 polymer/monomer fluorescence ratio. Red represents the fluorescence ratio of JC-1 polymer, and blue represents the fluorescence ratio of JC-1 monomer. **(C)** Different treatment groups, JC-1 red fluorescence/green fluorescence ratio statistical histogram. **(D)** Detection of secretion levels of oxidative factors ROS and MDA and antioxidant factors SOD and GSH in cells of different treatment groups. *P < 0.05, **P < 0.01, ***P < 0.001.

More and more evidences show that reactive oxygen species (ROS) are involved in the pathogenesis of multiple cochlear injuries, especially the apoptosis of SGNs induced by reactive oxygen species (Xiong et al., 2011; Zhuang et al., 2018). The protection of neurons from oxidative damage depends on the up-regulation of antioxidant response mediated by Nrf2/ARE. The redox-sensitive transcription factor Nrf2 and the anti-oxidant ARE response element dependent genes (GST, HO-1, SOD, etc.) play a vital role in the regulation of oxidative stress in cochlear SGNs. We detected the expression of Nrf2 and downstream HO-1 and NQO1 in SGSs after ouabain treatment. The results showed that ouabain treatment significantly inhibited the expression of Nrf2, HO-1 and NQO1 in SGSs, and was time-dependent (Figures 5A–C). However, we found that the addition of shikonin pretreatment can alleviate the inhibition of Nrf2/ARE signal pathway activity caused by ouabain, and significantly increase the expression of Nrf2 (Figure 6A). Subsequently, we tested the changes in mitochondrial membrane potential and oxidative stress levels. The results show that ouabain promotes the depolarization of mitochondrial membrane potential, while shikonin can significantly alleviate the sharp increase in mitochondrial depolarization caused by ouabain (Figures 6B,C). In addition, shikonin pretreatment can also significantly reduce the high levels of reactive oxygen species (ROS) and malondialdehyde (MDA) in SGNs caused by ouabain, and increase the levels of superoxide dismutase (SOD) and reduced glutathione (GSH) expression and secretion (Figure 6D). Therefore, shikonin may activate the Nrf2-ARE signal pathway inhibited by ouabain, thereby reducing the oxidative stress level of SGNs.

### Knockdown of Nrf2 Reduces the Protective Effect of Shikonin on Spiral Ganglion Neuron Cells Differentiation

**FIGURE 7:**
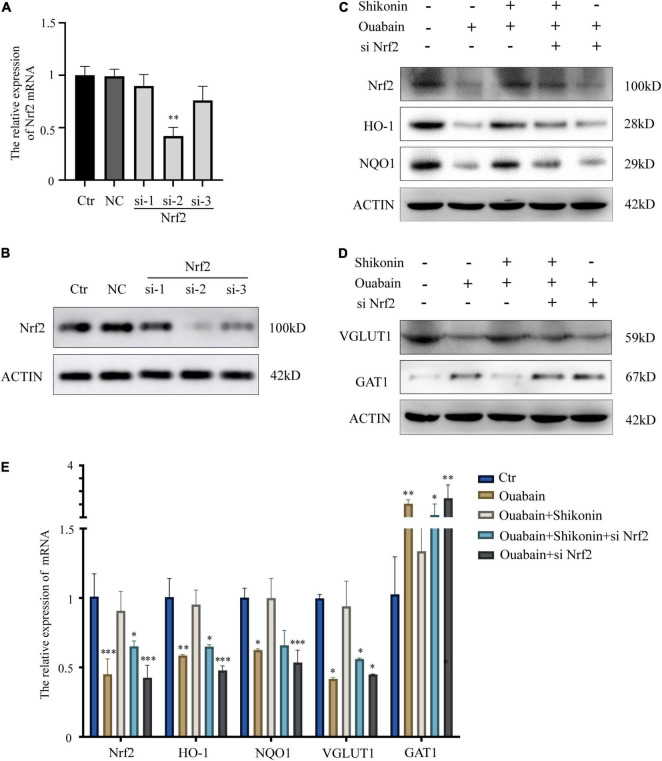
Knockdown of Nrf2 reduces the protective effect of shikonin on SGNs differentiation. **(A)** The specificity and efficiency of siRNA against Nrf2 were tested by quantitative PCR. **(B)** The knockout efficiency of Nrf2 siRNA was detected at the protein level by Western blot. SGNs were transfected with Nrf2 siRNA for 24 h, pretreated with shikonin, and then treated with ouabain for another 22 h. To detect the expression changes of Nrf2/ARE signal pathway (Nrf2, HO-1, and NQO1) protein **(C)** and mRNA **(E)** in SGNs. As well as the protein **(D)** and mRNA **(E)** expression changes of glutamatergic neurons VGLUT1 and GABAergic neurons GAT1. *P < 0.05, ^**^P < 0.01, ^***^P < 0.001.

Next, to explore the role of Nrf2/ARE signal pathway in the function of SGNs, we used specific small interfering RNA to knock down the expression of Nrf2 in SGNs. According to the knockout efficiency of small interfering RNA (Figures 7A,B), we choose siNrf2-2 for follow-up experiments. The results of the study show that knockdown of Nrf2 will significantly affect the expression of downstream factors HO-1 and NQO1 (Figures 7C,E). More importantly, the mitigation effect of shikonin on the inhibition of Nrf2, HO-1 and NQO1 expression caused by ouabain will also be lost due to the knockdown of Nrf2 (Figures 7C,E). In addition, the knockdown of Nrf2 will also hinder the regulation of shikonin on the differentiation of SGNs after ouabain injury. The suppression of VGLUT1 expression and the increase of GAT expression caused by ouabain improved by shikonin are lost (Figures 7D,E). The above results indicate that Nrf2 plays an irreplaceable role in the protection and regulation of shikonin on the function of SGNs.

### Shikonin Has Protective Effects on Ouabain-Induced Hearing Damage in Mice

**FIGURE 8:**
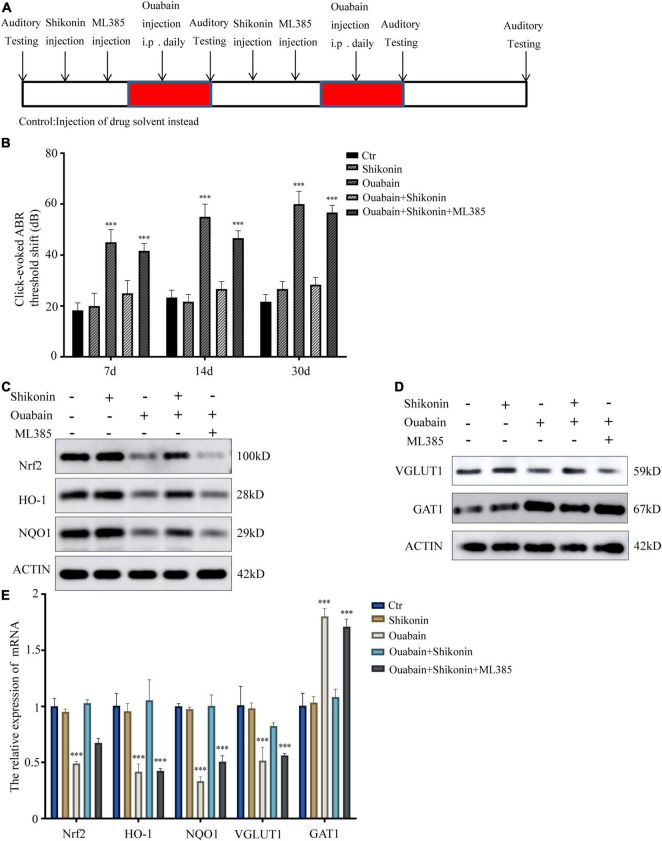
Shikonin has protective effects on ouabain-induced hearing damage in mice. **(A)** Experimental procedure of drug injection in mice. The arrow represents the drug injection point. The red bars represent the time period of continuous drug injection. The mice in the control group were injected with the same amount of normal saline or drug solvent at the same time point. **(B)** The statistical histogram of the hearing brainstem response of mice at 7, 14, and 30 days after drug injection (*n* = 3). After 14 days of drug treatment, detect the expression of Nrf2/ARE signal pathway (Nrf2, HO-1, and NQO1) protein **(C)** and mRNA **(E)** in mouse SGN tissue protein. And detect the protein **(D)** and mRNA **(E)** expressions of glutamatergic neurons VGLUT1 and GABAergic neurons GAT1. ^***^P < 0.001.

Considering that small interfering RNA transfection in mice *in vitro* has low efficiency and difficult operation, we choose Nrf2/ARE signaling pathway inhibitor ML385 to inhibit the activity of Nrf2/ARE signaling pathway in mice. ML385 can directly interact with Nrf2 protein and bind to the Neh1 binding region of Nrf2, thus preventing the binding of Nrf2-Mafg complex to the promoter ARE sequence and reducing transcriptional activity (Singh et al., 2016). Shikonin, inhibitors and ouabain need to be treated multiple times. In order to prevent multiple surgical damages from affecting the hearing and life status of mice, we chose intraperitoneal injection. The mouse hearing damage model and the time point of drug pretreatment are shown in Figure 8A. The control group was replaced by injection of the same amount of drug solvent. We tested the hearing changes of mice at 7, 14, and 30 days after drug injection. The results showed that the hearing loss of mice in the shikonin pretreatment group was significantly less than that of the mice without shikonin pretreatment, and Nrf2/ARE signaling pathway inhibitor ML385 pretreatment will greatly reduce the protective effect of shikonin on the hearing of mice (Figure 8B). Subsequently, we detected the expression of Nrf2/ARE signaling pathway proteins in mouse cochlea SGNs. The results showed that the expression of Nrf2, HO-1 and NQO1 in mouse cochlea SGNs in the ouabain treatment group was significantly reduced. Shikonin pretreatment can alleviate the ouabain-induced Inhibition of Nrf2/ARE signaling pathway protein expression (Figures 8C,E). The expression of VGLUT1 in the cochlea SGNs of mice in the ouabain treatment group was significantly reduced, and the expression of GAT1 was significantly increased. Shikonin pretreatment can significantly improve the differentiation imbalance of glutamatergic and GABAergic neurons in SGNs caused by ouabain (Figures 8D,E). In addition, the inhibition of the Nrf2/ARE signaling pathway will weaken the regulation of shikonin on the expression of differentiation proteins in mouse SGNs (Figures 8D,E). In summary, shikonin has a protective effect on ouabain-induced hearing damage in mice, and this protective effect depends on the activation of the Nrf2/ARE signaling pathway.

## Discussion

The spiral ganglion (SGN) is the first transfer station in the cochlear auditory pathway. It converts the external sound mechanical stimulus received by the hair cells into electrical signals, and then transmits them to the auditory center of the brain, completing the conversion of sound from the physical world into the perceptual world (Reijntjes and Pyott, 2016). The survival of spiral ganglion Schwann cells (SGSs) has attracted more and more attention of researchers due to the protective effect of the secreted factors on spiral ganglia. At present, the degeneration and death of SGN in the inner ear mainly include primary neurodegeneration and secondary neurodegeneration (the consequence of loss of hair cells) (Kujawa and Liberman, 2015; Guo J. et al., 2019). Due to the stimulation of ototoxic drugs, SGNs and SGSs in the cochlea gradually undergo morphological changes after being injured, including axons retreat, auditory nerve endings degenerate into neuron cell bodies, and finally neurons die. Schwann cells myelinize the peripheral processes, the initial part of the central axon, and the neuronal cell bodies of spiral ganglion neurons (SGNs) (Rangaraju et al., 2009), and the development, maintenance and regeneration of peripheral nerves involve dynamic interactions between neurons and Schwann cells (Sugiura and Lin, 2011). The search for drugs that can alleviate SGNs and SGSs retrograde degeneration caused by ototoxic drugs and lead to cell death is very important for hearing protection.

In clinical drug applications, many drugs have greatly limited their clinical applications due to ototoxic side effects (such as ouabain, cisplatin, etc.) (Liu et al., 2016; He et al., 2017), and many researchers have tried to reduce their ototoxicity (Gao et al., 2019; Zhang et al., 2019). Ouabain is a cardiac glycoside that can block the activity of Na+/K+-ATPase through specific binding (Lelkes, 2020). Studies have shown that ouabain can specifically damage SGNs when acting on the round window of the cochlea, but has little effect on the morphology and function of sensory epithelial hair cells and stria vascularis cells. Therefore, this study established a mouse model of neurological deafness with ouabain. In this study, we chose to inject ouabain through the intraperitoneal cavity of the mouse instead of directly administering the mouse by opening the round window in the ear to establish a mouse model of deafness. On the one hand, considering that the pretreatment of therapeutic drugs requires multiple administrations, the increased frequency of operations will have a greater impact on the survival of mice; On the other hand, it is more suitable to establish a non-acute deafness model and make the deafness model more stable. Another advantage is that the operation is simple and easy. Our research results show that ouabain can significantly increase the hearing threshold of mouse ABR and significantly damage the morphology of SGSs (Figure 1).

In the cochlea, the balance maintenance of inhibitory and excitatory neurotransmitters is one of the key regulatory factors for the physiological functions of the cochlea. Neurons in the cochlea can be divided into glutamatergic neurons and GABAergic neurons according to the neurotransmitters they produce (Wang et al., 2015; Huang et al., 2019). Among them, glutamatergic neurons produce excitatory neurotransmitters, and GABAergic neurons inhibit glutamatergic impulses (Huang et al., 2018). Our research results show that ouabain inhibits the differentiation of neurons into glutamatergic neurons, promotes the differentiation of GABAergic neurons, reduces the generation of excitatory nerve impulses, and reduces the sensitivity of the cochlea to sound stimulation (Figure 2). Ouabain affects VGLUT1 and GAT1 expression through regulation of neural cell differentiation but not cell proliferation.

Considering that the death of mouse cochlea SGSs is often caused by increased levels of autophagy, apoptosis, and oxidative stress. We tested the levels of autophagy and apoptosis in SGSs cells after ouabain treatment, and the results showed that with the gradual increase in the concentration of ouabain treatment, the levels of apoptosis and autophagy in SGSs increased (Figure 1). Although there are reports that shikonin is an ideal antioxidant, anti-apoptotic agent, and anti-inflammatory agent, it has not been reported whether shikonin has a protective effect on ototoxicity of drugs (such as ouabain). We speculate that shikonin has a protective effect on SGCs damage. In order to verify our hypothesis, we pretreated with shikonin before ouabain stimulated SGNs or SGSs. The results showed that shikonin pretreatment can significantly increase the survival rate of SGNs and SGSs after ouabain treatment, and reduce the apoptosis, autophagy and oxidative stress levels of SGNs and SGSs (Figures 3, 6). In addition, shikonin pretreatment also improves the potential of SGNs to differentiate into glutamatergic, promotes the generation and transmission of excitatory nerve impulses, and prepares for improving the sensitivity of sound stimulation (Figure 4).

Antioxidant drugs can reduce the damage of SGCs and protect SGCs from degeneration. Nrf2 regulates the transcription of genes encoding antioxidants, detoxification enzymes, and other vital factors for cell survival by regulating the antioxidant response element (ARE) (Hoshino et al., 2011). Studies have shown that intraperitoneal injection of rosmarinic acid can activate the Nrf-2/heme oxygenase-1 (HO-1) signaling pathway in the cochlea of rats, thereby reducing the production of superoxide and lipid peroxidation caused by noise (Fetoni et al., 2015). In order to explore whether the increase in oxidative stress levels in degraded SGCs after ouabain treatment is regulated by the Nrf2/ARE signaling pathway, we analyzed the expression of Nrf2, HO-1 and NQO1 after ouabain treatment, which are widely recognized Nrf2/ARE signals Pathway markers. The results showed that ouabain significantly inhibited the activity of the Nrf2/ARE signaling pathway (Figure 5), and Shikonin can alleviate ouabain’s damage to SGNs through the activation of the Nrf2/ARE signaling pathway (Figure 6); Knockdown or inhibition of Nrf2 can hinder the protective effect of shikonin on SGNs damage (Figures 7, 8).

## Conclusion

To conclude our research, we have proved through *in vitro* and *in vivo* experiments that ouabain can damage the growth and differentiation of SGNs, and promote cell apoptosis by increasing the level of oxidative stress in SGNs and SGSs. As an antioxidant, shikonin has a significant protective effect on ototoxicity caused by ouabain. In the cochlea, SGCs damage is regulated by complex signaling pathways. Shikonin can inhibit the increase of oxidative stress and abnormal cell differentiation caused by ouabain by activating the activity of the Nrf2/ARE signaling pathway. For the protection of ototoxic drugs-induced neurological degeneration of SGCs, shikonin may be a better candidate antioxidant drug.

## Data Availability Statement

The original contributions presented in the study are included in the article/Supplementary Material, further inquiries can be directed to the corresponding authors.

## Ethics Statement

The animal study was reviewed and approved by the Ethics Committee of the Affiliated Hospital of Shandong First Medical University. Written informed consent was obtained from the owners for the participation of their animals in this study.

## Author Contributions

HD drafted the important content of the manuscript and explained it, and carried out rigorous conception and design of the subject. XZ, MX, and LS carried out a detailed analysis of the data in the article. YW, NG, and HH carried out the collection of clinical samples. HY, ZT, FZ, and PZ conducted experimental operations. MZ and CL provided the subject ideas and careful proofreading of the manuscript. All authors contributed to the article and approved the submitted version.

## Conflict of Interest

HD was employed by the company Qilu Pharmaceutical Co., Ltd. The remaining authors declare that the research was conducted in the absence of any commercial or financial relationships that could be construed as a potential conflict of interest.

## Publisher’s Note

All claims expressed in this article are solely those of the authors and do not necessarily represent those of their affiliated organizations, or those of the publisher, the editors and the reviewers. Any product that may be evaluated in this article, or claim that may be made by its manufacturer, is not guaranteed or endorsed by the publisher.

## Supplementary Material

Supplementary Figure 1 Ouabain can induce autophagy and apoptosis in SGNs cells. Western Blot was used to detect the changes of apoptosis and autophagy protein levels in SGNs after ouabain treatment.
