# Supplementary figures and images for: Shikonin Attenuates Cochlear Spiral Ganglion Neuron Degeneration by Activating Nrf2-ARE Signaling Pathway
Source: Front Mol Neurosci. 2022 Feb 24;15:829642. doi: 10.3389/fnmol.2022.829642 (PMC8908960; doi:10.3389/fnmol.2022.829642)

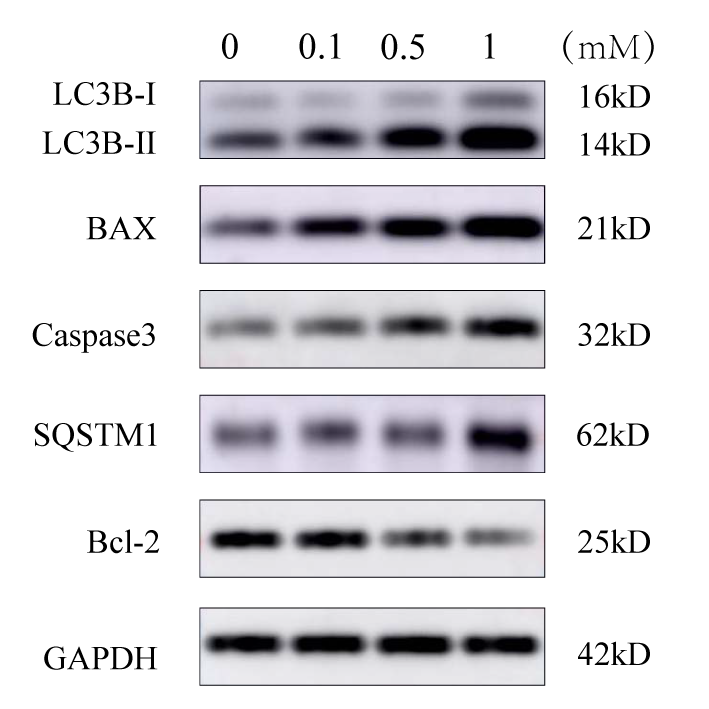

Supplement: Supplementary Figure 1 — Ouabain can induce autophagy and apoptosis in SGNs cells. Western Blot was used to detect the changes of apoptosis and autophagy protein levels in SGNs after ouabain treatment. [file Image_1.TIF]
